# The Circular RNA circXPO1 Promotes Tumor Growth *via* Sponging MicroRNA-23a in Prostate Carcinoma

**DOI:** 10.3389/fonc.2021.712145

**Published:** 2021-07-27

**Authors:** Haoyan Chen, Ping Zhang, Bo Yu, Jinlong Liu

**Affiliations:** ^1^Department of Pharmacy, Tongren Hospital, Shanghai Jiao Tong University School of Medicine, Shanghai, China; ^2^Zhangjiang Institute, Fudan University, Shanghai, China

**Keywords:** circXPO1, microRNA-23a, prostate cancer, therapeutic target, real-time PCR

## Abstract

It has been shown that circular RNA XPO1 (circXPO1) is involved in cancer (e.g., lung adenocarcinoma and osteosarcoma) progression by sponging microRNAs. Nevertheless, the role of circXPO1 and its interaction with microRNAs in prostate cancer remains unknown. In this study, the results of quantitative real-time PCR showed that circXPO1 levels were dramatically increased in human prostate cancer tissue and cell lines compared with those in normal tissue and cell line. Furthermore, cell proliferation, colony formation, and cell invasion assays showed that circXPO1 promoted the malignant behavior of pancreatic cells *in vitro*. Mechanistically, bioinformatics prediction, a dual-luciferase reporter assay, and pull-down assay suggested that circXPO1 physically targets miR-23a and negatively regulates its expression in pancreatic cancer cells. miR-23a mimics and inhibitors effectively reversed the effects of circXPO1 on the malignant behavior of prostate cancer cells *in vitro*. Consistent results were observed in the xenograft tumor model. In conclusion, circXPO1 promotes prostate cancer progression *via* targeting miR-23a, thus suggesting the circXPO1/miR-23a axis can be used as a potential therapeutic target for prostate cancer treatment.

## Introduction

Prostate cancer is the second most common cancer and the fifth leading cause of cancer-related death in men worldwide (1). For patients with primary prostate cancer, prostatectomy is commonly used. Although patients with early-stage diseases generally have good prognoses, up to 30% of cases suffer relapses within 5–10 years posttreatment. Moreover, the 5-year relative survival is around 30% for patients with distant metastasis (2, 3). Inflammation is an initial process in which cells are trafficked into the tumor microenvironment by specific cytokines called chemokines. This recruitment is complex and involves multiple subsets of white blood cells with anti-cancer and anti-cancer functions in the progress from benign prostatic enlargement to prostate cancer (4, 5). Androgen deprivation therapy is the first-line therapy for recurrent or metastatic prostate cancer. However, some patients may develop castration resistance (6). Thus, it is of urgent importance to identify novel therapeutic strategies for the treatment of prostate cancer.

Circular RNAs (circRNAs) are endogenous non-coding RNA characterized by a covalently closed loop structure lacking the 5’ cap and 3’ poly-A tail. circRNAs have an important role in the regulation of gene functions and the pathogenesis of human diseases, including cancer (7, 8). CircRNAs act as microRNA sponges to protect the target genes from microRNA-mediated mRNA cleavage. The circRNA/microRNA interaction modulates target gene expression and affects cancer cell proliferation, differentiation, invasion, and metastasis (9, 10). Previous studies have shown that circRNA/microRNA interaction is involved in prostate cancer progression. For example, circMYLK promotes prostate cancer progression by targeting mir-29a (11). Moreover, circSMARCA5 is upregulated in prostate cancer and promotes cancer cell proliferation (12). However, the function of circRNAs and their interactions with microRNAs in prostate cancer remains largely unknown.

CircXPO1 (circBase ID: hsa_circ_0001016, alias: hsa_circ_001767) is a novel circRNA that is derived from back-spliced exportin 1 (XPO1), a well-known cancer therapeutic target (13, 14). Studies have shown that circXPO1 is highly expressed in lung adenocarcinoma, osteosarcoma, and gallbladder cancer (13, 15, 16). In lung adenocarcinoma and osteosarcoma, circXPO1 expression, which is positively correlated with XPO1 expression, negatively affects patients’ overall survival, thus suggesting that circXPO1 promotes cancer progression and may be used as a potential therapeutic target for cancer treatment. In osteosarcoma, circXPO1 sponges multiple microRNAs, including miR-23a-3p, miR-23b-3p, miR-23c, and miR-130a-5p, to upregulate XPO1 expression (15), which suggests that circXPO1 facilitates cancer progression by acting as a competing endogenous RNA. Nevertheless, the role of circXPO1 in prostate cancer and the underlying mechanism remain unknown.

In this study, we explored the role of circXPO1 in prostate cancer growth and progression *in vitro* and *in vivo*. We found that circXPO1 regulates the malignant behavior of prostate cancer cells by targeting miR-23a. Our findings suggest that circXPO1 could be used as a novel, potential therapeutic target for prostate cancer treatment.

## Materials and Methods

### Patients and Sample Collection

This study was approved by the Ethics Committee of Fudan University Shanghai Cancer Center (No. 1608163; Shanghai, China). Written informed consent was obtained from each patient. Prostate cancer tissue and corresponding adjacent noncancerous tissue (> 5 cm from the tumor margin) were obtained from 48 patients (**Table 1**) with primary prostate carcinoma at Fudan University Shanghai Cancer Center. All tissue samples were immediately processed after surgical removal. Two experienced pathologists histologically confirmed the diagnosis.

**Table 1:** Detailed information of the 48 patients.

Patient ID	Gender	Age	Gleason Score	Grade	T	N	M	Clinical Stage
1815659	Male	74	3+4 = 7	2	T2	N0	M0	2B
1701493	Male	70	3+4 = 7	2	T2	N0	M0	2B
1705679	Male	65	3+4 = 7	2	T2	N0	M0	2B
1711389	Male	76	3+4 = 7	2	T2	N0	M0	2B
1712283	Male	65	3+4 = 7	2	T2	N0	M0	2B
1729272	Male	73	3+4 = 7	2	T2	N0	M0	2B
1615945	Male	57	3+4 = 7	2	T2	N0	M0	2B
1617235	Male	73	4+3 = 7	3	T2	N0	M0	2C
1645382	Male	77	4+3 = 7	3	T2	N0	M0	2C
1921049	Male	68	3+3 = 6	1	T2	N0	M0	3A
1647448	Male	76	4+3 = 7	3	T2	N0	M0	3A
1810237	Male	79	3+3 = 6	1	T4	N0	M0	3B
1436845	Male	78	3+3 = 6	1	T4	N0	M0	3B
1901662	Male	83	3+4 = 7	2	T3	N0	M0	3B
1802181	Male	81	3+4 = 7	2	T3	N0	M0	3B
1812607	Male	62	3+4 = 7	2	T3	N0	M0	3B
1830109	Male	76	3+4 = 7	2	T4	N0	M0	3B
1834984	Male	70	3+4 = 7	2	T3	N0	M0	3B
1732754	Male	63	3+4 = 7	2	T3	N0	M0	3B
1746937	Male	72	3+4 = 7	2	T3	N0	M0	3B
1626787	Male	68	3+4 = 7	2	T4	N0	M0	3B
1531534	Male	68	3+4 = 7	2	T3	N0	M0	3B
1915589	Male	69	4+3 = 7	3	T3	N0	M0	3B
1812902	Male	71	4+3 = 7	3	T4	N0	M0	3B
1813094	Male	72	4+3 = 7	3	T4	N0	M0	3B
1831974	Male	71	4+3 = 7	3	T3	N0	M0	3B
1735558	Male	71	4+3 = 7	3	T4	N0	M0	3B
1745899	Male	74	4+3 = 7	3	T3	N0	M0	3B
1748012	Male	64	4+3 = 7	3	T3	N0	M0	3B
1442076	Male	70	4+3 = 7	3	T3	N0	M0	3B
1737812	Male	72	3+5 = 8	4	T3	N0	M0	3B
1823731	Male	72	4+4 = 8	4	T4	N0	M0	3B
1824209	Male	75	4+4 = 8	4	T3	N0	M0	3B
1630336	Male	70	5+3 = 8	4	T3	N0	M0	3B
1708153	Male	72	4+5 = 9	5	T3	N0	M0	3C
1716752	Male	69	4+5 = 9	5	—	N0	M0	3C
1732705	Male	60	4+5 = 9	5	—	N0	M0	3C
1504663	Male	73	4+5 = 9	5	—	N0	M0	3C
1725134	Male	70	5+4 = 9	5	T3	N0	M0	3C
1726869	Male	61	5+4 = 9	5	T3	N0	M0	3C
1441266	Male	60	5+4 = 9	5	T3	N0	M0	3C
1726174	Male	67	5+5 = 10	5	T3	N0	M0	3C
1735625	Male	69	5+5 = 10	5	T4	N0	M0	3C
1644793	Male	62	3+3 = 6	1	—	N1	M0	4A
1833726	Male	78	3+4 = 7	2	—	N1	M0	4A
1851780	Male	73	3+4 = 7	2	T3	N1	M0	4A
1912914	Male	72	4+3 = 7	3	T3	N1	M0	4A
1817685	Male	78	4+3 = 7	3	T3	N1	M0	4A

### Cell Lines and Cell Culture

Human prostate cancer cell lines (PC-3, DU145, and 22RV1) and normal prostatic stromal myofibroblast cell line WPMY-1 were obtained from the American Type Culture Collection (Manassas, VA, USA) and maintained in Dulbecco’s modified Eagle’s medium supplemented with 10% fetal bovine serum (FBS; Gibco, Grand Island, NY) in a humidified atmosphere containing 5%CO_2_/95% air at 37°C.

### Overexpression and Knockdown of XPO1

PC-3 and DU145 cells were transfected with wild-type circXPO1- or circXPO1 mutant-overexpressing lentiviral vectors, small interfering RNA (siRNA) against circXPO1 (5’-CCATTCTTTGCTTCGCACTG-3’), miR-23a mimics, miR-23a mutant, miR-23a inhibitors, and corresponding negative control using Lipofectamine 3000 (Invitrogen, Carlsbad, CA, USA) according to the manufacturer’s instructions.

### Xenograft Mouse Model

The animal study was approved by the Ethics Committee of Fudan University Shanghai Cancer Center. All procedures were conducted following the guidelines of the National Institute of Health regarding the care and use of laboratory animals (NIH Publication No. 8023, revised 1978).

Female BALB/c nude mice aged 6-week-old were purchased from the China Academy of Sciences (Beijing, China) and maintained under pathogen-free conditions at Fudan University Shanghai Cancer Center. Mice were randomly divided into 4 groups and subcutaneously inoculated with 1 × 10^7^ PC-3 cells transfected with si-circXPO1, negative control siRNA (si-NC), si-NC + miR-23a inhibitor, or si-circXPO1 + miR-23a inhibitor into a single flank. The length and width of the tumors were measured every 5 days using a vernier caliper. The tumor volume was calculated using the following formula: *(length × width^2^)/2*. Mice were euthanized 25 days after inoculation. The tumors were immediately collected and weighed.

### Cell Viability Assay

PC-3 or DU145 cells were plated in 96-well plates and cultured overnight. Cells were transfected, as mentioned above. The Cell Counting Kit-8 (CCK8) assay (Dojindo Molecular Technologies, Kumamoto, Japan) was performed to determine the cell viability at 24, 48, or 72 h after transfection. Absorbance at 450 nm was measured using an automatic microplate reader (Infinite M200; Tecan, Grodig, Austria).

### Colony Formation Assay

Transfected PC-3 or DU145 cells were seeded in a 6-well plate at a density of 10^3^ cells/well and incubated at 37°C for 7 days. The cells were fixed with 4% paraformaldehyde and then stained with crystal violet, followed by colony counting. Images were acquired using a Zeiss microscope (Axio Observer, Zeiss, Germany) at magnification 10 ×. The experiments were repeated three times.

### Cell Migration and Invasion Assays

For cell migration assay, 1×10^5^ PC-3 or DU145 cells in 100 µL serum-free medium were added in the upper Transwell chamber (8.0-µm pore size; BD, San Jose, CA, USA). For the invasion assay, the upper chamber was coated with Matrigel. Medium containing 10% FBS was added to the lower chambers. After 24 h, the cells at the lower surface were fixed with methanol and stained with 1% crystal violet. Cells were counted, and images were acquired using a Zeiss microscope (Axio Observer, Zeiss, Germany) at magnification 10 ×.

### Quantitative Real-Time PCR (qRT-PCR)

Total RNA was isolated using the Ultraspec system (Biotecx, Houston, TX, USA) according to the manufacturer’s instructions. The cytoplasmic and nuclear RNA was isolated and purified using RNeasy Kits (Qiagen) following the manufacturer’s protocol. Quantitative PCR was performed using SYBR Green Fast Advanced Cells-to-CT Kit (Thermo Fisher) on an ABI PRISM 7900 Sequence Detector System (Applied Biosystems, Foster City, CA, USA). GAPDH or U6 was used as an internal reference. PCR reactions were performed in triplicate. Gene expression was quantified using the 2^−△△ct^ method. The primer sequences are summarized in **Table 2**.

**Table 2:** Primer sequences for quantitative real-time PCR.

Genes	Forward primer (5’–3’)	Reverse primer (5’–3’)
CircXPO1	CCAGTGCGAAGCAAAGAATGGC	CGCACTGGTTCCTTGGAAGA
GAPDH	GCGGGGCTCTCCAGAACATC	TCCACCACTGACACGTTGGC
MiR-23a	GGGGTTCCTGGGGATGGGATTT	TGGTGTCGTGGAGTCG
U6	CTCGCTTCGGCAGCACATATACT	ACGCTTCACGAATTTGCGTGTC

### Bioinformatics Prediction and Luciferase Reporter Assay

The potential microRNA targets of circXPO1 were predicted using miRanda (http://www.microrna.org/microrna/home.do). CircXPO1 with a wild-type or mutant miR-23a binding site was cloned into the psiCheck2 firefly luciferase vector (Promega, Madison, WI, USA). PC-3 or DU145 cells were cotransfected with the luciferase vectors and miR-23a mimics or negative control mimics. The Firefly and Renilla luciferase were detected using a dual luciferase assay kit (Promega), 48 h after transfection. The experiments were performed in triplicate.

### Fluorescence In Situ Hybridization (FISH)

For tissue samples, after fixing with 4% formaldehyde, the paraffin-embedded sections (5-μm thick) were prepared and dehydrated in a graded series of alcohol (100, 95, 85, and 75%). For cell samples, cells were fixed with 4% formaldehyde for 1 h, followed by incubation with the pre-block buffer for 15 min. Then, the slides or cells were incubated with circXPO1 probe (GTGCGAAGCAAAGAATGG) for 2 h at 37°C. The nuclei were counterstained with 4,6-diamidino-2-phenylindole. The images were acquired using a fluorescence microscope (LSM800; Zeiss, Germany).

### MicroRNA Pull-Down Assay

A total of 1×10^7^ cells were transfected with miR-24a or miR-24a mutant, as mentioned above. At 24 h after transfection, cells were harvested, washed with phosphate-buffered saline, and lysed with lysis buffer. Cells were then incubated with biotinylated-circXPO1 or GAPDH probes (Simo Biotech, Hangzhou, Zhejiang, China) at room temperature for 2 h. The biotin-coupled RNA complex was pulled down by incubating the cell lysates with streptavidin magnetic beads (Simo Biotech). The beads were washed with lysis buffer, and Trizol LS (Thermo Fisher Scientific, Waltham, MA, USA) was used to recover the RNA. The abundance of circXPO1 was determined by qRT-PCR.

### Argonaute2 (AGO2) Immunoprecipitation

RNA-binding protein immunoprecipitation (RIP) was performed using an anti-AGO2 antibody (PB1030, BOSTER) and the RIP assay kit (RIP-12RXN, Sigma Aldrich) following the manufacturer’s protocol. Briefly, cells were collected and lysed using RIP lysis buffer. The cell lysates were then incubated with RIP buffer containing magnetic beads conjugated to the anti-AGO2 antibody or negative control IgG. The samples were incubated with proteinase K to digest proteins, and then the immunoprecipitated RNA was isolated. The purified RNA was subjected to qRT-PCR to detect the presence of miR-23a and circXPO1. The total RNA was used as the input control.

### Hematoxylin and Eosin and Immunohistochemical (IHC) Staining

The tumor tissue samples were fixed with 4% paraformaldehyde for 24 h. The paraffin-embedded sections (4-μm thick) were prepared and dehydrated in a graded series of alcohol. The sections were stained with hematoxylin and eosin and mounted with neutral gum.

The protein expression of Ki67 was determined by IHC staining. Briefly, the paraffin-embedded sections were dewaxed in xylene and dehydrated in ethanol, followed by antigen retrieval with EDTA. The sections were blocked with 5% bovine serum albumin, and then incubated with anti-Ki67 (ab15580, 1:200, Abcam). Detection of the antigen-antibody complex was performed using a secondary antibody (ab97040,1:2000, Abcam) and a DAB detection kit (34002, Thermo Fisher). Images were acquired using an Axio Vert.A1 microscope (Zeiss, Germany) at magnification 20 ×.

### Statistical Analysis

Data were expressed as the means ± standard deviation. Statistical analysis was conducted using SPSS 22.0 (SPSS, Chicago, IL, USA). Differences between groups were compared using a one-way analysis of variance, followed by a Student *t*-test. A *P* value < 0.05 was considered statistically significant.

## Results

### CircXPO-1 Levels Are Increased in Human Prostate Cancer Tissue and Cell Lines

**Figure 1:**
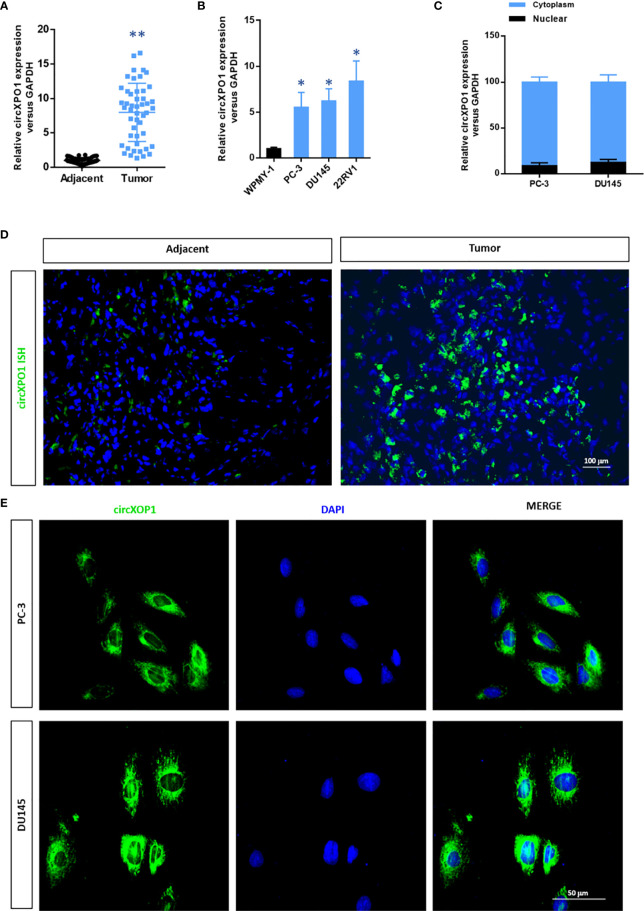
CircXPO1 expression was upregulated in prostate cancer tissue and cell lines. **(A)** Quantitative real-time PCR (qRT-PCR) was performed to determine circXPO-1 expression in pancreatic cancer tissue and adjacent normal tissue. Data are expressed as the means ± standard deviation (SD). **P < 0.01 *vs.* adjacent normal tissue; n = 48. **(B)** qRT-PCR was performed to determine circXPO-1 expression in normal prostatic stromal myofibroblast cell line WPMY-1 and pancreatic cancer cell lines (PC-3, DU145, 22RV1). Data are expressed as the means ± SD. *P < 0.05 *vs.* WPMY-1; n = 3. **(C**–**E)** qRT-PCR **(C)** and fluorescence *in situ* hybridization assay **(D, E)** were conducted to examine the nuclear and cytoplasmic expression of circXPO-1.

To investigate the expression of circXPO1 in prostate carcinoma, we examined the expression of circXPO1 in prostate cancer tissue and cell lines. The results of qRT-PCR showed that prostate cancer tissue samples had remarkably higher circXPO1 levels than the matched adjacent normal tissue samples (**Figure 1A**). Similar data were observed in prostate cancer cell lines PC-3, DU145, and 22RV1 that showed dramatically increased circXPO1 levels compared with normal prostatic stromal myofibroblast cell lines WPMY-1 (**Figure 1B**); yet, the highest expression was seen in the cytoplasm of PC-3 and DU145 cells (**Figure 1C**). FISH assay further confirmed the predominant expression of circXPO-1 in prostate cancer tissue and the cytoplasm of prostate cancer cells (**Figures 1D, E**).

### CircXPO-1 Promotes Cell Proliferation, Colony Formation, and Invasion in Prostate Cancer Cells

**Figure 2:**
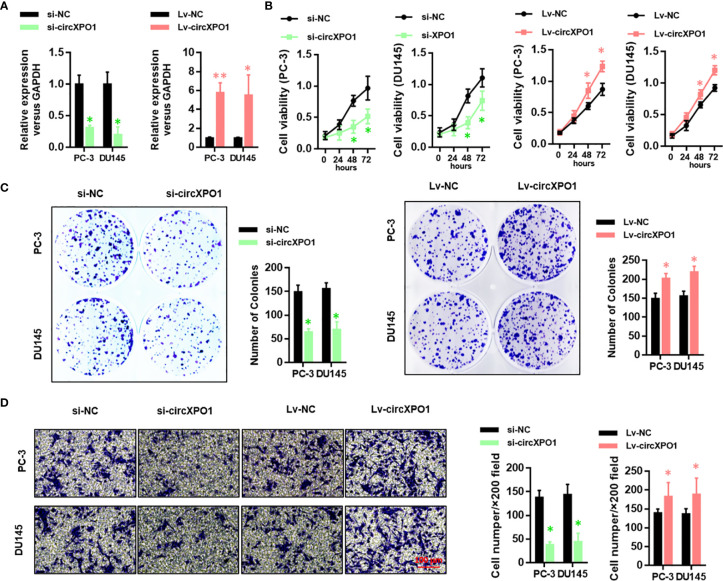
CircXPO-1 promoted cell proliferation, colony formation, and invasion in prostate cancer cells. **(A)** PC-3 and DU145 cells were transfected with small interfering RNA against circXPO1 (si-circXPO1), negative control (si-NC), lentiviral vectors expressing circXPO1 (Lv-circXPO1), or Lv-NC. qRT-PCR was performed to evaluate the knockdown and overexpression efficiency. **(B)** PC-3 cells were transfected as indicated. CCK-8 assay was conducted to measure cell viability at 0, 24, 48, and 72 h after transfection. **(C)** PC-3 and DU145 cells were transfected as indicated and incubated for 7 days. A colony formation assay was performed. **(D)** PC-3 and DU145 cells were transfected as indicated. Transwell assay was performed to evaluate the invasive ability of cells. Data are expressed as the means ± SD. *P < 0.05, **P < 0.01, *vs.* NC; n = 3.

Next, we performed gain- and loss-of-function assay to explore the function of circXPO1 in prostate cancer (**Figure 2A**). circXPO1 silencing significantly inhibited cell proliferation (**Figure 2B**) and colony formation (**Figure 2C**), whereas circXPO1 overexpression promoted cell proliferation and colony formation in PC-3 and DU-145 cells compared with negative control. In addition, circXPO1 silencing dramatically inhibited cell invasion, whereas circXPO1 overexpression significantly promoted cell invasion in PC-3 and DU-145 cells (**Figure 2D**). These results indicate that circXPO-1 promotes the malignant behavior of prostate cancer cells.

### CircXPO-1 Targets miR-23a in Pancreatic Cancer Cells

**Figure 3:**
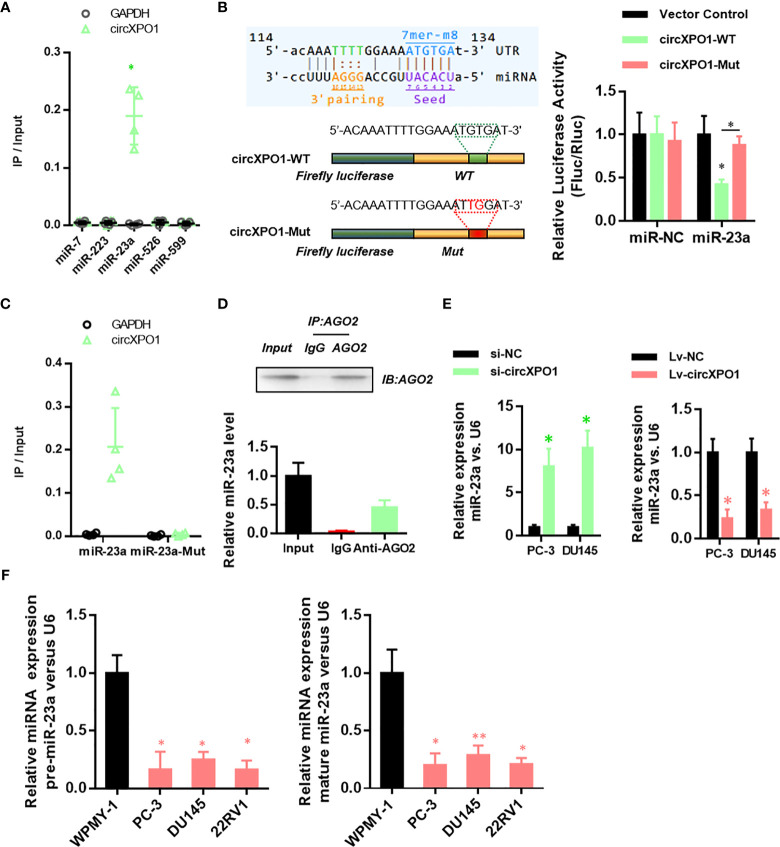
CircXPO-1 targeted miR-23a. **(A)** RNA pull-down was conducted to identify the target microRNA of circXPO-1. **(B)** DU145 cells were cotransfected with the luciferase vectors expressing the wild-type or mutant circXPO-1 and miR-23a mimics or negative control mimics. The luciferase signals were detected at 48 h after transfection. **(C)** RNA pull-down with wild-type or mutant miR-23a was performed to examine the interaction between miR-23a and circXPO1. **(D)** AGO2 immunoprecipitation was conducted to examine the enrichment of miR-23a and circXPO1 in the AGO2 complex. **(E)** qRT-PCR was performed to determine miR-23a levels in PC-3 or DU145 cells in response to circXPO1 knockdown or overexpression. *P < 0.05 *vs.* si-NC; n = 3. **(F)** qRT-PCR was conducted to determine miR-23a levels in normal prostatic cell line WPMY-1 and pancreatic cancer cell lines (PC-3, DU145, 22RV1). *P < 0.05, **P < 0.01, *vs.* WPMY-1; n = 3.

To reveal the underlying mechanism of circXPO1 in regulating the malignant behavior of prostate cancer cells, we sought to find the target microRNAs of circXOP-1. By analyzing the circXPO1 sequence using the miRanda database, we found potential binding sites for miR-7, miR-223, miR-23a, miR-526, and miR-599. The miRNA pull-down assay revealed that only miR-23a could bind to circXPO1 (**Figure 3A**). Dual-luciferase reporter assay showed that overexpression of miR-23a inhibited the luciferase activity of circXPO1 wild-type reporter, but not the luciferase activity of circXPO1 mutant reporter (**Figure 3B**). The miRNA pull-down assay further confirmed that circXPO1 was enriched with wild-type miR23a rather than miR-23a mutant (**Figure 3C**). Moreover, the AGO2 immunoprecipitation assay showed that both circXPO1 and miR-23a were enriched in the precipitated AGO2 complex (**Figure 3D**). These results suggest that circXPO1 physically interacts with miR-23a.

We further explored whether circXPO1 regulates miR-23a expression in pancreatic cancer cells. circXPO1 silencing dramatically enhanced miR-23a expression, whereas circXPO1 overexpression inhibited miR-23a expression, compared with negative control (**Figure 3E**). Furthermore, pancreatic cancer cell lines exhibited substantially lower levels of pre- and mature miR-23a than WPMY-1 cells (**Figure 3F**). Collectively, these results suggest that circXPO1 binds to miR-23a, acting as a miR-23a sponge in pancreatic cancer cells.

### circXPO-1 Promotes Cell Proliferation and Tumor Growth in Prostate Cancer by Sponging miR-23a

**Figure 4:**
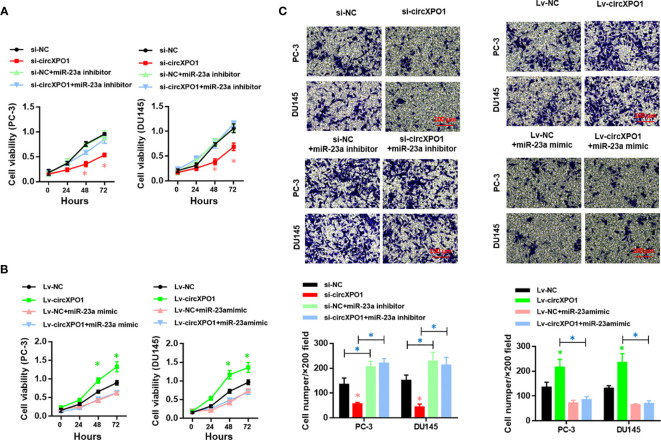
circXPO-1 promoted cell proliferation, migration, and invasion of prostate cancer cells by sponging miR-23a in *vitro*. PC-3 or DU145 cells were transfected as indicated. CCK-8 assay **(A, B)** and trans-well assay **(C)** were carried out to examine cell proliferation, migration, and invasion. Data are expressed as the means ± SD. *P < 0.05; n = 3.

Next, we sought to investigate whether circXPO-1 regulates the malignant behavior of pancreatic cancer cells *via* sponging miR-23a. As shown in **Figure 4A**, the miR-23a inhibitor effectively restored the proliferative abilities of circXPO-1-silenced PC-3 and DU154 cells. On the other hand, miR-23a mimic dramatically abolished the promoting effects of circXPO-1 overexpression on pancreatic cancer cell proliferation (**Figure 4B**). Similar results were observed after performing cell migration and invasion assays (**Figure 4C**).

**Figure 5:**
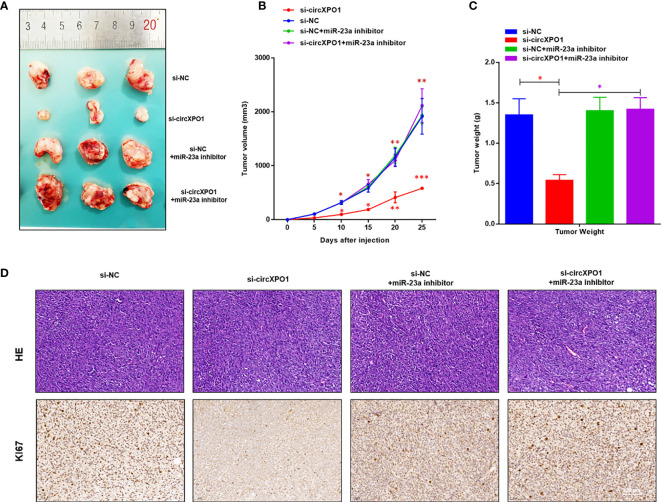
circXPO-1 promoted prostate tumor growth by sponging miR-23a in *vivo*. Mice were subcutaneously inoculated with 1 × 10^7^ PC-3 cells transfected with si-circXPO1, si-NC, si-NC + miR-23a inhibitor, or si-circXPO1 + miR-23a inhibitor into a single flank. **(A)** Images of the tumors. **(B)** The growth curve of the tumors. The tumor volume was calculated as (length × width^2^)/2. **(C)** Mice were euthanized 25 days after inoculation. The tumors were immediately collected and weighed. Data are expressed as the means ± SD. *P < 0.05, **P < 0.01, ***P < 0.001; n = 3. **(D)** The tumor tissue samples were subjected to Hematoxylin & eosin staining (upper panel) and immunohistochemical staining for Ki67 (lower panel).

We also established a xenograft tumor model in nude mice to investigate the function of circXPO1 *in vivo*. circXPO1 silencing resulted in considerably smaller tumor mass and decreased Ki67 levels in tumor tissue compared with negative control, which was entirely reversed by cotransfection with miR-23a inhibitor (**Figures 5A–D**). Collectively, these results suggest that circXPO-1 promotes cell proliferation and tumor growth in prostate cancer by sponging miR-23a.

## Discussion

This study demonstrated that circXPO1 levels were significantly increased in human prostate cancer tissue and prostate cancer cell lines compared with those in corresponding controls. We further revealed that circXPO1 promotes prostate cancer progression by sponging miR-23a. To the best of our knowledge, this is the first study that reported on the expression, function, and mechanism of circXPO1 in prostate cancer.

At present, serum prostate-specific antigen (PSA) remains the standard biomarker for the diagnosis and treatment of prostate cancer (17). However, due to the poor specificity of PSA test, it often leads to overdiagnosis and overtreatment. There is still no consensus on whether this screening test can significantly reduce the mortality of prostate cancer (18). In addition, the pathogenesis and molecular mechanisms of invasion and metastasis of prostate cancer are still unclear (19, 20). It is important to identify genetic drivers of prostate cancer so that new biomarkers can be developed to stratify the risk and aggressiveness of prostate cancer during screening. CircRNAs, which are widely expressed in mammalian cells, are resistant to RNase R degradation. Due to their unique loop structure, they are more stable biomarkers than lineal RNAs (11, 21). In the present study, the qRT-PCR analysis showed that circXPO1 was upregulated in human prostate cancer tissue and prostate cancer cell lines, thus suggesting that circXPO1 may serve as a biomarker for prostate cancer. The increased expression of circXPO1 in prostate cancer tissue and cell lines suggests that circXPO1 might promote prostate cancer progression. As expected, our data showed that overexpression of circXPO1 promotes cell proliferation, colony formation, and invasion, whereas knockdown of circXPO1 suppressed the malignant behavior of prostate cancer cells. In the prostate cancer xenograft mouse model, circXPO1 silencing resulted in considerably smaller tumor mass and decreased Ki67 levels in tumor tissue compared with the negative control. These results suggest that circXPO1 is a potential therapeutic target for prostate cancer treatment.

Considering the well-established interaction between circRNAs and microRNAs, we further identified potential microRNA targets of circXPO1 to investigate the mechanisms underlying its role in prostate cancer. Bioinformatics analysis demonstrated that circXPO1 harbored a miR-23a binding site. Dual-luciferase reporter assay, RNA FISH, and RNA pull-down assay further confirmed that circXPO1 physically targets miR-23a in prostate cancer cells. These findings suggest that circXPO1 and miR-23a might exhibit opposite expression patterns in prostate cancer tissue and exert opposite roles in prostate cancer progression. Indeed, Cai *et al.* found that miR-23a levels are decreased in prostate cancer cell lines and tumor tissue. They also found that low miR-23a levels are associated with poor prognoses of patients with prostate cancer and that MiR-23a inhibits prostate cancer cell migration and invasion both *in vitro* and *in vivo* (22). Similarly, Aghaee-Bakhtiari et al. have shown that miR-23a is significantly downregulated in prostate cancer cell lines and tissue samples (23). These findings suggest that miR-23a acts as a tumor suppressor in prostate cancer and that circXPO1 facilitates the progression of prostate cancer by competing and sponging miR-23a, which is consistent with our results.

As a robust regulator of gene expression, miR-23a targets a broad range of mRNAs in cancer cells by binding to the 3′-untranslated region (UTR), which in turn suppresses gene expression. For instance, miR-23a promotes colorectal cancer progression by targeting PDK4 (24). MiR-23a facilitates breast cancer metastasis by targeting CDH1 (25). MiR-23a acts as an oncogene in pancreatic carcinoma by targeting FOXP2 (26) and promotes tumor progression by targeting SETD2 in various carcinoma (27). In prostate cancer, miR-23a can target the APK and JAK/STAT pathways, the PAK6-LIMK1 pathway, and mitochondrial glutaminase (22, 23, 28). This study did not investigate the downstream target genes of the circXPO1/miR-23a axis, which will be addressed in future studies.

In conclusion, our study identified circXPO1 as a novel tumor promoter in prostate cancer. CircXPO1 facilitates prostate cancer progression by sponging miR-23a, thus serving as a potential therapeutic target for prostate cancer treatment.

## Data Availability Statement

The original contributions presented in the study are included in the article/supplementary material. Further inquiries can be directed to the corresponding authors (miguelboyu@msn.cn).

## Author Contributions

(I) Conception and design: BY. (II) Administrative support: BY. (III) Provision of study materials or patients: PZ. (IV) Collection and assembly of data: PZ, HC. (V) Data analysis and interpretation: HC. (VI) Manuscript writing: All authors. All authors contributed to the article and approved the submitted version.

## Conflict of Interest

The authors declare that the research was conducted in the absence of any commercial or financial relationships that could be construed as a potential conflict of interest.

## Publisher’s Note

All claims expressed in this article are solely those of the authors and do not necessarily represent those of their affiliated organizations, or those of the publisher, the editors and the reviewers. Any product that may be evaluated in this article, or claim that may be made by its manufacturer, is not guaranteed or endorsed by the publisher.
